# Multidetector Computed Tomographic Evaluation of the Normal Characteristics of the Thymus in the Pediatric Population

**DOI:** 10.5334/jbsr.2971

**Published:** 2022-11-17

**Authors:** Edis Çolak, Behzat Özkan

**Affiliations:** 1 Department of Radiology, University of Health Sciences Dr. Behçet Uz Child Disease and Pediatric Surgery Training and Research Hospital, Izmir, Turkey; 2 Department of Pediatric Endocrinology and Metabolism, University of Health Sciences Dr. Behçet Uz Child Disease and Pediatric Surgery Training and Research Hospital, Izmir, Turkey

**Keywords:** Thymus, mediastinum, children, computed tomography

## Abstract

**Objectives:**

The objective of the present study was to determine the morphologic features and measurements of the normal thymus on contrast-enhanced multi-detector computed tomography (MDCT) in subjects from the newborn period up to 18 years of age.

**Materials and Methods:**

The MDCT scans obtained from 464 children with a mean age ± SD of 8.43 ± 5.60 years were retrospectively re-evaluated. The shape, margins, side predominance, density, and measurements of the thymic gland were defined for each age group.

**Results:**

A triangular thymic shape with a middle location and straight lateral contours were the most frequently seen morphologic features in children. The mean anteroposterior and transverse diameter of the thymus was 17.32 ± 4.58 and 29.99 ± 11.42 mm, respectively. The mean values for the width and thickness were 20.66 ± 5.36 and 15.15 ± 6.76 mm for the right thymic lobe, respectively; and 26.14 ± 7.85 and 14.91 ± 5.51 mm for the left, respectively. The transverse diameter of the thymus and thymic lobe dimensions decreased significantly with age, however, the anteroposterior diameter of the thymus was not significantly associated with age. Girls had higher mean thymic attenuation values compared to boys, however, this gender difference was not statistically significant (63.8 ± 22.4 HU vs. 60.1 ± 25.3, p = 0.164).

**Conclusion:**

Our study provides a better understanding of the normal thymic appearances in children that can aid in accurate diagnosis and avoid unnecessary, costly, and invasive interventions.

## Introduction

Computed tomography (CT) imaging evaluation of a normal thymus can be challenging since the morphologic features and the size of the thymus may differ even between children of the same age [1]. This is an important point, especially in patients who have a history of lymphoma, germ cell tumor, or sarcoma where it is difficult to decide whether anterior mediastinal soft tissue density demonstrates normal thymus or a space-occupying neoplasm. Thus, in suspicious cases, further diagnostic imaging techniques and even invasive procedures may be performed to exclude pathology [2,3].

As no established guidelines are available, the CT interpretation of a normal thymus in children is mainly based on expert opinion. To the authors’ knowledge, the studies regarding morphologic appearance, dimensions, and CT attenuation values of the normal thymus were most recently conducted in 1982, 1987, and 2000, respectively using the earlier generations of CT scanners and a small number of children [4,5,6]. However, to date, no previous study has used the current multi-detector CT (MDCT) technology to define the appearance of the normal thymus in a relatively large sample of children stratified by age and gender.

The objective of the current study was to determine the morphologic features and measurements of the normal thymus on MDCT in subjects from the newborn period up to 18 years of age.

## Material and Methods

### Study population

The study protocol was approved by the Institutional Medical Research Ethics Committee following the guidelines of the Declaration of Helsinki (protocol number: 2022/688). A descriptive cross-sectional and retrospective study design was employed for this research.

Over a period of five years from January 2016 to December 2021, 1,610 pediatric patients underwent thoracic MDCT examination in our University Hospital for various reasons including traumatic and nontraumatic thoracic emergencies and congenital or acquired pulmonary diseases. The exclusion criteria were defined as follows: subjects above 18 years of age [n = 28]; mediastinal or neck infection [n = 24]; lung infection (diffuse parenchymal infection, fungus ball, hydatid cyst) [n = 251]; mediastinal trauma (pneumomediastinum, hemomediastinum) [n = 17]; mediastinal mass (lymphadenopathy, teratoma, lymphoma, thymoma, neuroblastoma) [n = 146]; mediastinal distortion caused by thoracic or abdominal pathologies (pneumothorax, pulmonary lobe atelectasis, congenital lobar pulmonary emphysema, cardiomegaly, diaphragmatic hernia) [n = 84]; severe chest wall abnormalities [n = 14]; ectopic thymus [n = 5]; diseases associated with thymic enlargement (Graves’ disease, inflammatory bowel disease, cystic fibrosis) [n = 24]; conditions associated with thymic regression (mediastinal radiation therapy, chemotherapy, use of corticosteroids) [n = 142]; previous mediastinal or neck surgery [n = 9]; patients with hematologic and oncologic disorders [n = 375]; and CT investigations with poor image quality [n = 27].

Following these exclusion criteria, a total of 464 patients remained in the study population.

### CT protocol

The thoracic contrast-enhanced MDCT examinations were obtained from the apex to the base of the chest using a 32-detector-row CT scanner (SOMATOM go. Now, Siemens, Erlangen, Germany) with the following parameters: matrix, 512 × 512; tube rotation time, 0.3–0.6 s; pitch, 1; section thickness, 3 mm; and intersection gap, 1 mm. A non-ionic iodinated contrast medium (300–320 mg I/mL), was administered intravenously at a dose of 1–2 ml/kg and an injection rate of 0.8–2.5 ml/s. Using the bolus tracking method and a region of interest, which was placed in the descending aorta, the arterial phase CT scan was initiated at a threshold of 140 Hounsfield units (HU). In order to keep the radiation as low as reasonably achievable (ALARA), acquisitions were performed at tube currents of 20–150 mA and a tube voltage of 80–120 kV depending on the patient’s weight. CT images were evaluated at window settings appropriate for mediastinum (window level, 30–40; and window width, 300–400) on a Picture Archiving and Communication System (PACS) workstation (Virtual Place Raijin, AZE Ltd., Tokyo, Japan).

### Analysis of the imaging findings

**Figure 1:**
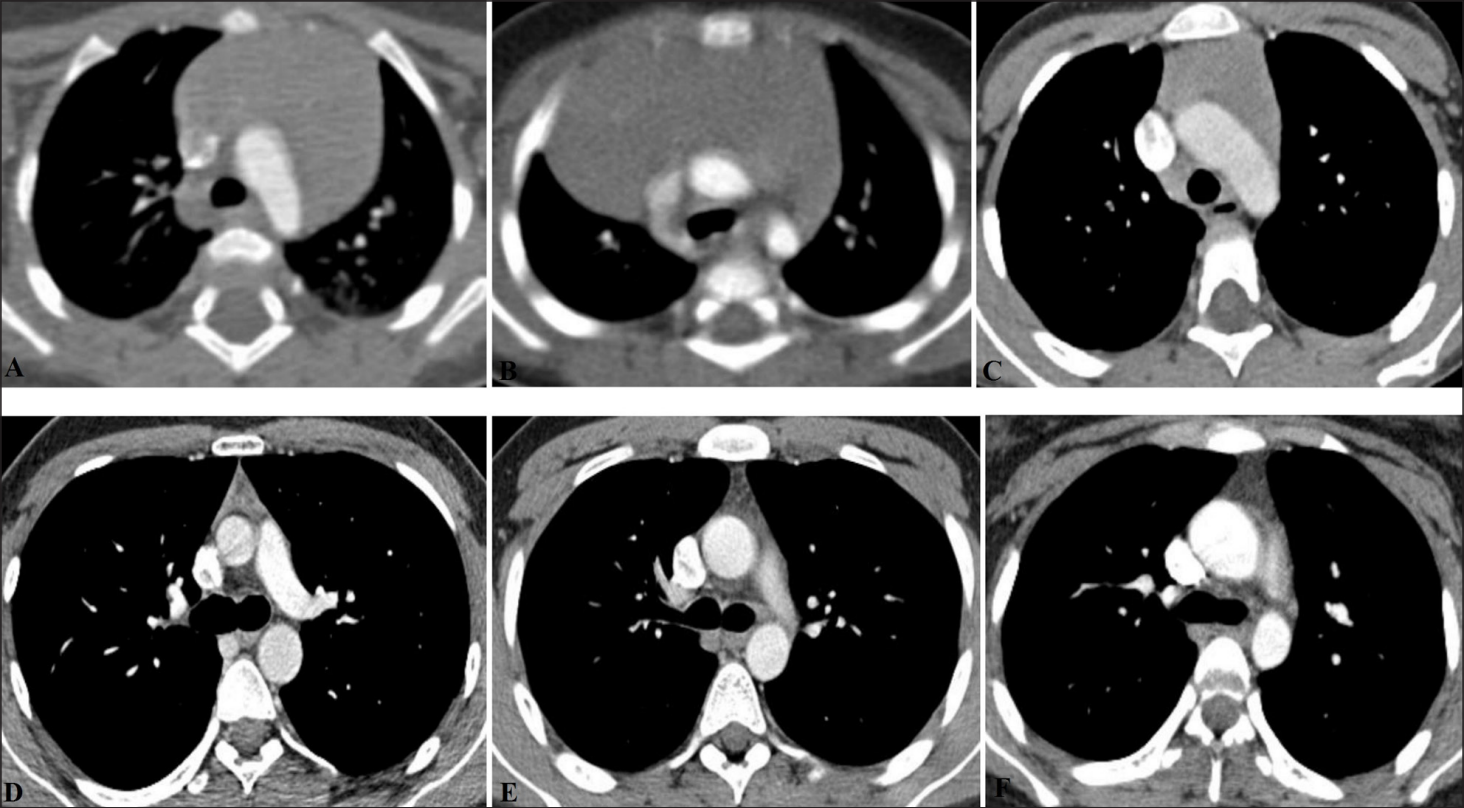
The axial thoracic contrast-enhanced computed tomography images present the morphologic features of the thymus in children. **(A)** 1 year old girl; round-oval shape; biconvex margins; predominantly left-sided location; Score 3, mainly soft-tissue attenuated thymus. **(B)** 4 months old boy; round-oval shape; biconvex margins; predominantly right-sided location; Score 3, mainly soft-tissue attenuated thymus. **(C)** 5 years old boy; quadrilateral shape; mixed (right straight, left convex) margins; middle location; Score 3, mainly soft-tissue attenuated thymus. **(D)** 15 years old boy; triangular shape; straight margins; middle location; Score 2, nearly half fatty and half soft-tissue attenuated thymus. An example of a manually delineated free-hand region of interest (ROI) for measuring the CT attenuation value is presented. **(E)** 17 years old boy; triangular shape; straight margins; middle location; Score 1, mainly fatty thymus. **(F)** 18 years old boy; triangular shape; biconcave margins; middle location; Score 0, complete fatty thymus.

The assessment of the thymus was based on qualitative and quantitative approaches. In the first step, the shape, margins, and the side predominance of the thymus were described on three-dimensional CT images. The thymic shape was characterized as ‘round-oval’ (Figure 1A, B), ‘quadrilateral’ (Figure 1C), and ‘triangular’ (Figure 1D, F). The thymic margins were defined as ‘biconvex’ (Figure 1A, B), ‘biconcave’ (Figure 1F), and ‘straight’ (Figure 1D, E). The combination of concave and convex, concave and straight, or convex and straight borders was classified as ‘mixed’ (Figure 1C). The location of the thymic gland in the anterior mediastinum was defined in relation to the midline as midline (Figure 1C, D, E, F), predominantly right-sided (Figure 1B), and predominantly left-sided (Figure 1A).

The fat content in the thymic gland was scored using a four-point scale (0 to 3) scoring system as previously described by Ackman et al. [7] as follows: Score 0, complete involution and fatty replacement of the thymus (Figure 1F); Score 1, mainly fatty thymus (Figure 1E); Score 2, nearly fifty percent of fatty and fifty percent of the soft-tissue attenuated thymus (Figure 1D); and Score 3, mainly soft-tissue attenuated thymus (Figure 1A, B, C) [7].

The mean thymic CT attenuation values in Hounsfield Units (HU) were measured on axial images using a manually delineated free-hand region of interest (ROI) covering the maximum area of the gland and avoiding the surrounding mediastinal fat, large blood vessels, motion artifacts, and partial volume effects (Figure 1D) [8,9].

**Figure 2:**
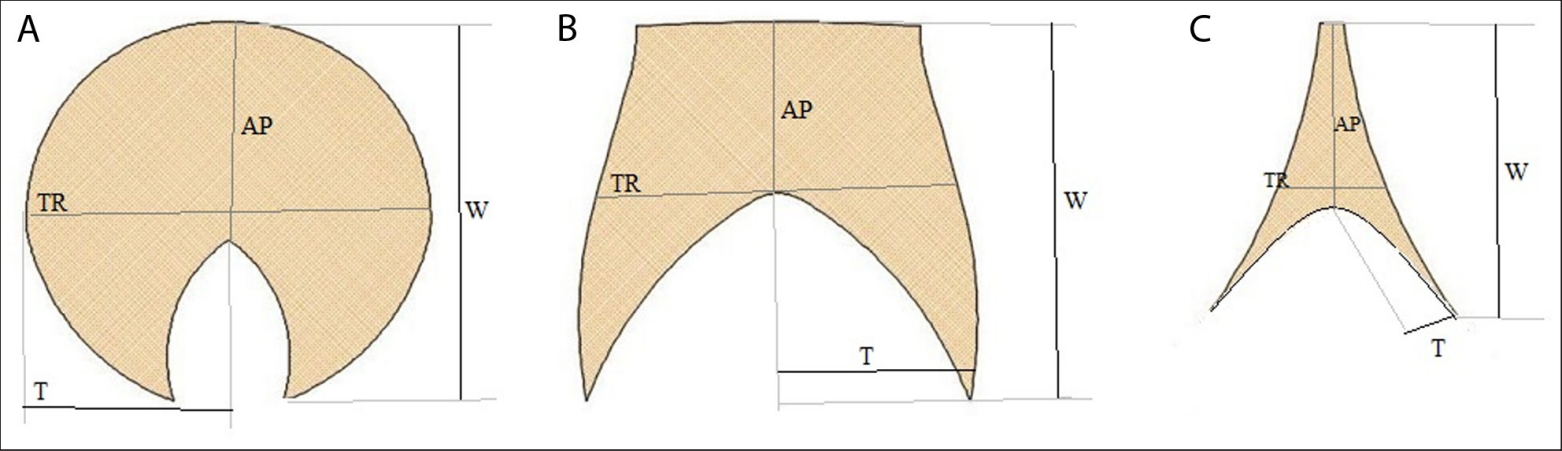
The graph shows the measurements of the maximum anteroposterior (AP) and transverse (TR) diameter of the thymus; maximum width (W) and thickness (T) of the thymic lobes in the **(A)** round-oval, **(B)** quadrilateral, and **(C)** triangular thymic shape.

The measurements were performed, as previously described, on a single axial CT image showing the largest area of the thymic gland [5,7,10]. The maximum anteroposterior (AP) diameter of the thymus was measured at the midline at its thickest part dividing the gland into the right and left lobes. The maximum transverse diameter of the thymus was determined by measuring the widest distance between the outer margins of the thymic lobes. The maximal thymic lobe width was estimated by measuring the longest distance from the midline to the farthest lateral margin of each lobe. The maximal lobe thickness was measured perpendicular to the long axis of each lobe (Figure 2).

The image analysis was performed by a single European Board of Radiology – certified radiologist who was blinded to the age and gender of the patients.

### Statistical analysis

Distributions of continuous variables were assessed for normality using the Skewness-Kurtosis test and Kolmogorov-Smirnov test. Normally distributed data were presented as mean ± standard deviation (SD) and compared by one-way analysis of variance (ANOVA) test followed by post hoc analyses. Skewed distributed data were expressed as median [interquartile range (IQR) 25 – 75] and analyzed by the Wilcoxon rank-sum test and Kruskal-Wallice test. Categorical variables were compared by the Chi-square test and summarized using frequencies and percentages.

After logarithmic transformation of non-normal distributed data, linear regression analyses adjusted for age and gender were performed to assess the relationship between thymic measurements and thymic CT attenuation values over time and age groups.

Data were analyzed statistically using SPSS software, version 20.0 (SPSS Inc., Chicago, IL). P-values less than 0.05 were regarded as statistically significant.

## Results

### Demographic data

The study group included 266 (57.3%) boys and 198 (42.7%) girls with a mean age ± SD of 8.43 ± 5.60 years (age range 0 – 18 years). Children were stratified into six groups according to their age: Group 1 (0 – 12 months, n = 24); Group 2 (1 – 3 years, n = 94); Group 3 (4 – 6 years, n = 83); Group 4 (7 – 10 years, n = 72); Group 5 (11 – 14 years, n = 104); and Group 6 (15 – 18 years, n = 87). The age groups were chosen according to the classification and definition of medical subject headings (MeSH) (i.e., infants, toddlers, preschool children, primary school-age children, secondary school-age children, and adolescents).

### Morphologic features of the thymus on CT

A quadrilateral shaped thymus (201/464, 43.3%) was the most frequently seen morphologic feature on CT in children, followed by triangular (169/464, 36.4%) and round-oval (94/464, 20.3%). A round-oval thymus was more common in younger children with a mean age ± SD of 3.02 ± 3.23 years. A triangular thymus was more often seen in older children aged 11 years and above. These age-related differences in thymic shape proved to be statistically significant (p < 0.0001).

The majority of children had straight (228/464, 49.1%), followed by biconvex (109/464, 23.5%), and mixed (106/464, 22.8%) thymic margins. The biconcave (21/464, 4.6%) borders were less commonly seen. Among the mixed thymic margins, 50% (53/106) of the study group had a combination of right straight-left convex and 33% (35/106) had right convex – left straight lateral contours. The thymus with biconvex margins was most commonly found in small children aged between 0 and 3 years. The thymus with straight contours was observed in nearly all subjects aged 15 years and above. The differences in thymic margins among age groups were statistically significant (p = 0.001).

The thymus was located at the midline in 266 (57.3%) and showed a predominance on the left side in 134 (28.9%), and on the right side in 64 (13.8%) children. The midline position of the thymus was more frequent among children older than 11 years of age. The right-sided thymus was common in infants aged 0–12 months. The age-related differences in the thymic side were statistically significant (p < 0.0001).

The shape, margins, and the side predominance of the thymus did not show differences among gender (p = 0.958, p = 0.059, p = 0.101, respectively).

The morphologic features of the thymus according to age groups are summarized in Table 1.

**Table 1:** Distribution of the shape, margins, and side predominance of the thymus according to age.


MORPHOLOGIC FEATURES OF THE THYMUS		GROUP 1 (0–12 MONTHS) N (%)	GROUP 2 (1–3 YEARS) N (%)	GROUP 3 (4–6 YEARS) N (%)	GROUP 4 (7–10 YEARS) N (%)	GROUP 5 (11–14 YEARS) N (%)	GROUP 6 (15–18 YEARS) N (%)	p-VALUE

**Shape**	**round-oval**	14 (3%)	52 (11.2%)	16 (3.4%)	5 (1.1%)	7 (1.5%)	0 (0.0%)	p^a^ < 0.0001

	**quadrilateral**	9 (1.9%)	38 (8.2%)	51 (11%)	37 (8%)	56 (12.1%)	10 (2.2%)	p^a^ < 0.0001

	**triangular**	1 (0.2%)	4 (0.9%)	16 (3.4%)	30 (6.5%)	41 (8.8%)	77 (16.6%)	p^a^ < 0.0001

**Margins**	**biconvex**	15 (3.2%)	48 (10.3%)	18 (3.9%)	10 (2.2%)	18 (3.9%)	0 (0.0%)	p^b^ < 0.0001

	**biconcave**	2 (0.4%)	2 (0.4%)	5 (1.1%)	2 (0.4%)	6 (1.3%)	4 (0.9%)	p^b^ = 0.489

	**straight**	2 (0.4%)	18 (3.9%)	31 (6.7%)	43 (9.3%)	54 (11.6%)	80 (17.2%)	p^b^ < 0.0001

	**mixed**	5 (1.1%)	26 (5.6%)	29 (6.2%)	17 (3.7%)	26 (5.6%)	3 (0.6%)	p^b^ < 0.0001

**Side predominance**	**midline**	8 (1.7%)	39 (8.4%)	38 (8.2%)	44 (9.5%)	61 (13.1%)	76 (16.4%)	p^c^ < 0.0001

	**right-sided**	12 (2.6%)	19 (4.1%)	9 (1.9%)	6 (1.3%)	16 (3.4%)	2 (0.4%)	p^c^ = 0.002

	**left-sided**	4 (0.9%)	36 (7.8%)	36 (7.8%)	22 (4.7%)	27 (5.8%)	9 (1.9%)	p^c^ < 0.0001


Abbreviations: mixed margins, a combination of concave and convex, concave and straight, or convex and straight margins; Data are expressed as number (n) and percentage (%); P^a^ – values for comparing each shape between age groups; P^b^ – values for comparing each margin between age groups; p^c^ – values for comparing each side predominance between age groups; p – values were obtained using the Chi-square test; p – values <0.05 were considered significant.

### Thymic attenuation assessment

The estimation of thymic attenuation was made via subjective and objective assessment. The majority of children in our study had a mainly soft-tissue attenuated thymus (Score 3) (391/464, 84.3%), followed by half fatty and half soft-tissue attenuated (Score 2) (56/464, 12.1%) and mainly fatty thymus (Score 1) (14/464, 3%). The complete fatty replacement of the thymus (Score 0) was observed in only 3 (0.6%) subjects. The thymic attenuation score did not show a significant gender difference (p = 0.271).

The mean thymic CT attenuation values were 61.7 ± 24.2 (range, – 40.2 – 104.6) HU. In general, girls had higher mean thymic attenuation values (63.8 ± 22.4 HU) compared to boys (60.1 ± 25.3 HU), and this gender difference was not statistically significant (p = 0.164). There was a good correlation between subjective thymic scoring and objective measurements of the CT attenuation values [F (1, 462) = 665,179, p < 0.0001, R^2^ = 0.590]. With increasing age, the fatty content of the thymus increased, while the thymic CT attenuation values diminished significantly (p = 0.002).

The distribution of the thymic density according to age is described in Table 2.

**Table 2:** Distribution of the thymic density according to age.


THYMIC ATTENUATION ASSESSMENT		GROUP 1 (0–12 MONTHS)	GROUP 2 (1–3 YEARS)	GROUP 3 (4–6 YEARS)	GROUP 4 (7–10 YEARS)	GROUP 5 (11–14 YEARS)	GROUP 6 (15–18 YEARS)	P-VALUE

**Thymic score**	**Score 1**	0 (0.0%)	0 (0.0%)	0 (0.0%)	0 (0.0%)	0 (0.0%)	3 (0.6%)	p = 0.006

	**Score 2**	0 (0.0%)	0 (0.0%)	0 (0.0%)	0 (0.0%)	0 (0.0%)	14 (3.0%)	p < 0.0001

	**Score 3**	0 (0.0%)	0 (0.0%)	0 (0.0%)	5 (1.1%)	13 (2.8%)	38 (8.2%)	p < 0.0001

	**Score 4**	24 (5.2%)	94 (20.3%)	83 (17.9%)	67 (14.4%)	91 (19.6%)	32 (6.9%)	p < 0.0001

**CT attenuation values (HU)**	**Male**	73.7 ± 8.4 (61.2–89.3)	75.4 ± 11.8 (53.4–104.6)	73.4 ± 13.0 (22.9–90.2)	67.1 ± 13.1 (22.1–90.3)	56.2 ± 19.8 (6–93.2)	25.0 ± 26.7 (-40.2–71.7)	p^a^ = 0.029

	**Female**	70.8 ± 10.2 (58.2–81.7)	74.8 ± 11.8 (51.1–97.5)	72.4 ± 9.2 (58.1–94.6)	66.8 ± 13.5 (42.1–88.6)	63.8 ± 17.6 (20.1–93.5)	39.2 ± 33.8 (-27.6–98.1)	p^b^ = 0.213

	**Total**	73.2 ± 8.6 (58.2–89.3)	75.2 ± 11.7 (51.1–104.6)	72.9 ± 11.2 (22.9–94.6)	67.0 ± 13.2 (22.1–90.3)	59.9 ± 19.1 (6.0–93.5)	30.9 ± 30.5 (–40.2–98.1)	p^c^ = 0.002


Abbreviations: Score 0, complete fatty replacement; Score 1, mainly fatty thymus; Score 2, 50% of fatty and 50% of the soft-tissue attenuated thymus; Score 3, mainly soft-tissue attenuated thymus; Data are expressed as number (n) and percentage (%); mean ± standard deviation (SD), range (minimum to maximum value); p – values for comparing each thymic score between age groups; p^a^ – values for comparing CT attenuation values among boys between age groups; p^b^ – values for comparing CT attenuation values among girls between age groups; p^c^ – values for comparing CT attenuation values between age groups; p – values were obtained using the Chi-square test and ANOVA – post hoc Bonferroni test; p – values <0.05 were considered significant.

### Thymic gland dimensions

The mean anteroposterior (AP) and transverse diameter of the thymus was 17.32 ± 4.58 (range, 6.22 – 35.45) and 29.99 ± 11.42 (range, 7.65 – 71.70) mm, respectively. The mean values for the width and thickness were 20.66 ± 5.36 (range, 5.45 – 42.06) and 15.15 ± 6.76 (range, 1.83 – 38.47) mm for the right thymic lobe, respectively; and 26.14 ± 7.85 (range, 6.00 – 55.27) and 14.91 ± 5.51 (range, 3.50 – 40.00) mm for the left, respectively.

**Figure 3:**
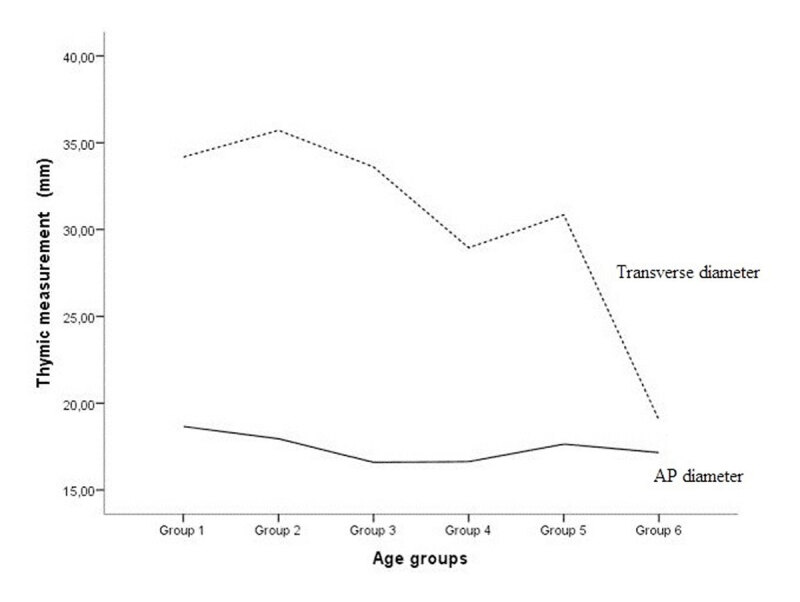
The graph shows the relationship between the estimated marginal means of the anteroposterior (AP) and transverse diameter of the thymus according to age groups.

Although insignificant, the width of the left thymic lobe showed dominance over the right lobe in both males and females (26.6 vs. 21.0 and 25.5 vs. 20.2, p = 0.185; respectively). The thymic gland was 1,5 mm wider in girls compared to boys; however, these changes did not reach the significant value (30.8 vs. 29.3, p = 0.160; respectively). The transverse diameter of the thymus and thymic lobe dimensions decreased significantly with age (p < 0.01 for all). The AP diameter of the thymus did not decrease significantly with age (p = 0.750) (Figure 3).

Table 3 shows the distribution of the thymic gland dimensions by gender and age category.

**Table 3:** The mean diameters of the thymic gland according to age and gender.


THYMIC GLAND DIMENSIONS (MM)		GROUP 1 (0 – 12 MONTHS)	GROUP 2 (1 – 3 YEARS)	GROUP 3 (4 – 6 YEARS)	GROUP 4 (7 – 10 YEARS)	GROUP 5 (11 – 14 YEARS)	GROUP 6 (15 – 18 YEARS)	p-VALUE

**Right thymic lobe width**	**M**	22.3 ± 4.8	23.0 ± 5.3	19.7 ± 4.3	19.9 ± 5.4	21.2 ± 6.3	19.8 ± 5.6	p^a^ = 0.053

	**F**	20.7 ± 2.7	20.5 ± 4.3	19.6 ± 4.0	19.7 ± 3.8	22.4 ± 6.6	18.1 ± 4.8	p^b^ = 0.448

	**T**	22.0 ± 4.5	22.1 ± 5.1	19.6 ± 4.1	19.8 ± 4.8	21.8 ± 6.4	19.1 ± 5.3	p^c^ = 0.011

**Right thymic lobe thickness**	**M**	18.1 ± 7.0	19.1 ± 8.2	16.1 ± 6.3	14.9 ± 6.0	14.3 ± 5.2	9.5 ± 5.3	p^a^ < 0.0001

	**F**	15.3 ± 2.5	17.8 ± 5.6	17.1 ± 5.0	14.8 ± 7.1	17.4 ± 8.2	8.9 ± 4.2	p^b^ < 0.0001

	**T**	17.6 ± 6.5	18.5 ± 7.3	16.6 ± 5.7	14.9 ± 6.4	15.8 ± 7.0	9.2 ± 4.9	p^c^ < 0.0001

**Left thymic lobe width**	**M**	25.7 ± 6.3	27.6 ± 7.7	29.2 ± 5.7	27.3 ± 5.9	27.3 ± 7.9	22.9 ± 9.0	p^a^ = 0.001

	**F**	23.5 ± 9.4	26.9 ± 7.6	25.5 ± 7.3	27.0 ± 6.9	26.5 ± 8.6	21.7 ± 8.1	p^b^ = 0.279

	**T**	25.3 ± 6.7	27.3 ± 7.6	27.3 ± 6.8	27.3 ± 7.9	26.9 ± 8.3	22.4 ± 8.6	p^c^ < 0.0001

**Left thymic lobe thickness**	**M**	16.5 ± 5.1	17.7 ± 5.4	16.0 ± 4.0	14.0 ± 4.4	13.9 ± 4.9	9.8 ± 4.4	p^a^ < 0.0001

	**F**	16.7 ± 2.5	17.1 ± 4.4	17.9 ± 4.2	14.1 ± 5.0	16.0 ± 6.6	10.5 ± 4.3	p^b^ < 0.0001

	**T**	16.5 ± 4.7	17.5 ± 5.1	17.0 ± 4.2	14.0 ± 4.6	14.9 ± 5.8	10.1 ± 4.4	p^c^ < 0.0001

**Thymic gland AP diameter**	**M**	18.4 ± 4.0	18.8 ± 4.4	16.8 ± 4.1	16.2 ± 4.0	17.9 ± 5.4	17.7 ± 5.0	p^a^ = 0.283

	**F**	20.2 ± 3.4	16.6 ± 4.8	16.4 ± 3.7	17.3 ± 4.2	17.4 ± 5.3	16.4 ± 3.8	p^b^ = 0.794

	**T**	18.7 ± 3.9	18.0 ± 4.7	16.6 ± 3.9	16.6 ± 4.1	17.6 ± 5.4	17.2 ± 4.5	p^c^ = 0.750

**Thymic gland transverse diameter**	**M**	34.6 ± 11.4	36.2 ± 10.2	32.1 ± 9.3	29.0 ± 9.6	33.5 ± 13.1	18.8 ± 9.0	p^a^ < 0.0001

	**F**	32.0 ± 4.9	34.9 ± 9.2	35.0 ± 8.3	29.0 ± 11.2	30.8 ± 11.6	19.4 ± 7.7	p^b^ < 0.0001

	**T**	34.2 ± 10.5	35.7 ± 9.8	33.6 ± 8.9	29.0 ± 10.1	18.8 ± 9.0	19.0 ± 8.4	p^c^ < 0.0001


Abbreviations: AP, anteroposterior Data are expressed as mean ± standard deviation (SD); p^a^ – values for comparing each thymic dimension values among boys between age groups; p^b^ – values for comparing each thymic dimension values among girls between age groups; p^c^ – values for comparing each thymic dimension values between age groups; p – values were obtained using the Kruskal – Wallis test; p – values <0.05 were considered significant.

## Discussion

Numerous infectious, neoplastic, systemic inflammatory, and autoimmune diseases frequently involve the thymus in the form of an anterior mediastinal mass or diffuse enlargement [2,3]. As the thymus undergoes dynamic changes with age, familiarity with the CT imaging findings of the normal thymus in the pediatric population is essential for the radiologists to take the responsibility for defining the thymus as normal for age and thus, avoid unnecessary further images and biopsies [1,2,3]. Ultrasound is a non-invasive and radiation-free diagnostic imaging modality capable of identifying thymic tissue in cases of doubt or disagreement. A high-resolution linear probe is most useful to determine the normal thymus appearance, which is homogeneous with a ‘liver-like’ echotexture and multiple hyperechoic foci giving a ‘starry sky’ pattern [11]. Moreover, the normal thymus does not cause midline crossing of the mediastinal structures [11].

The most recent studies describing the CT characteristics of the pediatric thymic gland were published in 1982, 1987, and 2000 and were performed using the earlier generation of CT scanners and small sample sizes [4,5,6]. However, the MDCT images allow a precise assessment of the thymus, due to the higher contrast resolution, the lack of superimposition, and the possibility of three-dimensional and multiplanar reconstructions. However, to date, no previous study has used the current MDCT technology to define the normal thymus in a relatively large sample of children stratified by age and gender. The aim of the present study was to evaluate the morphologic features, CT attenuation values, and measurements of the normal thymus in children aged between 0 and 18 years.

Amour et al. [5] have reported the thymic shape and lateral margins by CT in 71 subjects in the age range of 0 – 19 years. They have stated that the thymus in children below the age of five years had a quadrilateral shape with biconvex lateral contours. These features were observed in 33% of children aged 5 – 10 years and 11% aged 10 – 15 years. Another CT study, conducted by Heiberg et al. [4] found that the mixed lateral thymic contours were most commonly seen among the study group of 40 children aged less than 20 years, followed by biconvex (9/40) and biconcave (5/40) contours. In the present study, a round-oval shape with biconvex margins was the most frequently seen thymic morphologic characteristic on CT in children younger than three years of age. These features were observed in 13% of children aged 4–6 years, 4% in children aged 7–14 years, and 0% in children above 15 years of age. A quadrilateral thymic shape with a middle location and straight lateral contours were most commonly seen in children aged between 4 and 14 years. Similar morphologic appearances were reported in approximately 10% of study subjects under 3 years and above 15 years of age. The remaining 15 years and older children had a middle located triangular thymus with straight contours which was in agreement with a previous study [5]. These features were identified in only 5% of children aged under 6 years and nearly 20% of children aged between 7 and 14 years. The reasons for the differences between the present work and previous reports might be closely related to racial and environmental factors in addition to the limited number of participants in the previous studies [4,5].

Sklair-Levy et al. [6] have assessed the attenuation of the normal thymus in 152 children aged between 0 and 14 years. The mean thymic attenuation value in infants aged below 1 year was 80.8 ± 5.7 HU and significantly higher in male infants. The remaining age groups had mean CT values between 78.5 ± 11.8 HU in 2-y-old and 56.4 ± 14 HU in 14-y-old children. These values did not differ significantly between the gender of the older children. Our results in 0 -– 14 year-old children were nearly 10% higher than those reported by Sklair-Levy et al. [6]. However, this study was performed without the use of intravenous contrast administration which might be the main reason for the discrepancy between the results. The attenuation of the thymus measured on contrast-enhanced CT by Heiberg et al. [4] was ranged between 28 – 185 HU in children up to 20 years. In our study the thymic CT attenuation values ranged between – 40.2 and 104.6 HU. The different characteristics of the CT scanners between the period of the previous study (1981) and ours might influence the results.

To date, only two studies including both children and adults have evaluated the thymic gland measurements [10,12]. In the study by Baron et al. [10] the mean values for the width and thickness in 6–19 years-old children were 20 ± 5.5 and 10 ± 3.9 mm for the right thymic lobe, respectively; and 33 ± 10.1 and 11 ± 4.0 mm for the left, respectively. Park et al. [12] have investigated the mean width and thickness measurements in 0- to 19 year-old subjects being 27.5 ± 7.7 and 12.0 ± 4.9 mm for the right thymic lobe, respectively; and 35.5 ± 9.4 and 16.8 ± 6.9 mm for the left, respectively. These studies should be interpreted with caution since they were comprised of a small sample size, ranging from 10 to 40 children, and performed with the earlier generation of CT scanners. However, the width of both thymic lobes in our children was nearly 5–10% lower, and the thickness was nearly 2–10% higher than those found in the previous studies [10,12].

The limitations of the present study need to be acknowledged. This study was performed retrospectively at a single center. Although the overall sample size was large, the number of infants in the Group 1 was relatively low (n = 24) compared to other groups. The inter-and intra-observer coefficient of variation was not investigated in this research. Finally, the interpretation of the results was restricted by the paucity of publications providing data on the subject, thus, future multicenter studies with a larger sample size are needed to validate our results.

## Conclusion

In conclusion, the present study demonstrates that a triangular thymic shape with a middle location and straight lateral contours are the most frequently seen morphologic features on CT in children. The transverse diameter of the thymus and thymic lobe dimensions decreased significantly with age, however, the anteroposterior diameter of the thymus was not significantly associated with age. There was a significant relationship between age and thymic CT attenuation values. Gender was not correlated with the thymic size and density. Our study provides a better knowledge of the normal spectrum of thymic appearances in the pediatric population which is important to prevent unnecessary invasive procedures and referrals to higher levels of healthcare.
